# Prevalence study of *Legionella spp*. contamination in ferries and cruise ships

**DOI:** 10.1186/1471-2458-6-100

**Published:** 2006-04-18

**Authors:** Antonio Azara, Andrea Piana, Giovanni Sotgiu, Marco Dettori, Maria Grazia Deriu, Maria Dolores Masia, Bianca Maria Are, Elena Muresu

**Affiliations:** 1 Hygiene and Preventive Medicine Institute, University of Sassari, Sassari, Italy

## Abstract

**Background:**

In the last years, international traffic volume has significantly increased, raising the risk for acquisition of infectious diseases. Among travel-associated infections, increased incidence of legionellosis has been reported among travellers.

Aim of our study was: to describe the frequency and severity of *Legionella *spp. contamination in ferries and cruise ships; to compare the levels of contamination with those indicated by the Italian ministerial guidelines for control and prevention of legionellosis, in order to assess health risks and to adopt control measures.

**Method:**

A prevalence study was carried out on 9 ships docked at the seaports of northern Sardinia in 2004. Water samples were collected from critical sites: passenger cabins, crew cabins, kitchens, coffee bars, rooms of the central air conditioning system. It was performed a qualitative and quantitative identification of *Legionella *spp. and a chemical, physical and bacteriological analysis of water samples.

**Results:**

Forty-two percent (38/90) water samples were contaminated by *Legionella *spp.. Positive samples were mainly drawn from showers (24/44), washbasins (10/22). *L. pneumophila *was isolated in 42/44 samples (95.5%), followed by *L. micdadei *(4.5%).

Strains were identified as *L. pneumophila *serogroup 6 (45.2%; 19 samples), 2–14 (42.9%), 5 (7.1%) and 3 (4.8%). *Legionella *spp. load was high; 77.8% of the water samples contained > 10^4 ^CFU/L.

Low residual free chlorine concentration (0–0,2 mg/L) was associated to a contamination of the 50% of the water samples.

**Conclusion:**

*Legionella *is an ubiquitous bacterium that could create problems for public health.

We identified *Legionella *spp. in 6/7 ferries. Microbial load was predominantly high (> 10^4 ^CFU/L or ranging from 10^3 ^to 10^4 ^CFU/L). It is matter of concern when passengers are subjects at risk because of *Legionella *spp. is an opportunist that can survive in freshwater systems; high bacterial load might be an important variable related to disease's occurrence.

High level of contamination required disinfecting measures, but does not lead to a definitive solution to the problem. Therefore, it is important to identify a person responsible for health safety in order to control the risk from exposure and to apply preventive measures, according to European and Italian guidelines.

## Background

Tourism has played an important role in economic growth in Developed and Developing Countries.

According to the data of the body representing the private sector in all parts of the travel and tourism industry worldwide, i.e. the World Travel and Tourism Council, travel and tourism industry contribute 10.9% of gross international product [1,2].

Tourism has contributed significantly towards the Italian economic development; the impact on economy is estimated at 350 billions euros in the next ten years [2].

However, an increased risk for acquisition of diseases, such as those related to infection, is inevitably associated with the international traffic volume, as well as with the modern high-speed transport (high disease burden in developing countries).

Among travel-associated infections, increased incidence of legionellosis, infection caused by the bacterium *Legionella *spp., has been reported among travellers; at least 48 species of *Legionella *have been identified, with 18 of these species linked to human diseases [3]. *L pneumophila *is the most frequent cause of human legionellosis.

Legionellosis refers to 2 distinct clinical syndromes, namely, Legionnaires disease, which most often presents as severe pneumonia accompanied by multisystemic disease, and Pontiac fever, which is an acute, febrile, self-limited, viral-like illness [4].

*Legionella *are gram-negative, rod-shaped bacteria that are ubiquitous in freshwater environments. Transmission occurs through aerosolization or aspiration of contaminated water [5]. Infection depends on the water contamination level by bacteria, host factors (advanced age, tobacco smoke, chronic degenerative diseases, state of immunodeficiency), and the virulence of the particular strain of *Legionella *(enhanced by protozoa- or *amoeba*-bacterium relationship).

Most cases of Legionellosis are sporadic but outbreaks can occur due to contamination of hot and cold water systems, evaporative condensers, spa pools/natural pools/thermal springs, respiratory therapy equipment. Biofilm formation can provide a means for survival and dissemination of *Legionella *bacterium [6,7].

Cases might be linked to nosocomial-lethality rate: 30–50%- or travel-associated infection (tourist accommodation, vessel, etc.).

Owing to the possibility of environmental exposure to the organism, legionellosis is a public health problem, investigated by numerous health organizations, such as the World Health Organization (WHO), European Working Group for *Legionella *Infections (E.W.G.L.I) [8], Italian Istituto Superiore di Sanità.

On this basis, to assess the impact of *Legionella *spp. contamination in vessels, Hygiene and Preventive Medicine Institute of the University of Sassari, Italy, involved in the surveillance of nosocomial legionellosis in the last years, together with Maritime Health Office of Porto Torres, Italy, undertook an epidemiological investigation of ferries and cruise ships.

It addressed two aims: – to describe the frequency and severity of *Legionella *spp. contamination; – to compare the levels of contamination with those indicated by the Italian ministerial guidelines for control and prevention of legionellosis, in order to assess health risks and to adopt control measures.

## Methods

The survey was carried out on 9 ships docked at the seaports of northern Sardinia in 2004; 7 ferries belonged to two Italian shipping companies while 2 cruise ships belonged one to an Italian shipping company and another come from Bahamas. Epidemiological investigation was divided into three distinct types of activity:

• selection of suspected contaminated areas and their critical sites; particularly, five different areas were identified: cabin where people sleep, cabin where crew sleep, kitchen, coffee bar, room of the central air conditioning system. Water samples were collected from showers, washbasins, kitchen sinks, evaporative condensers of air conditioning systems.

• water sampling: two samples were collected from every sampling site in a single time-point (total amount = 90; ten samples were collected from every ship, five for qualitative and five for quantitative identification of *Legionella *spp). The first water sample was collected for qualitative evaluation, without flaming and immediately after the tap was switched on; the second sample was collected for quantitative analysis, after the water ran for at least 5–10 minutes, being more representative of the water flowing in the system.

• qualitative and quantitative analysis of *Legionella *spp, according to methods described in the guidelines for prevention and control legionellosis [9-11]; furthermore, determination of water quality was carried out in order to enumerate total and fecal coliforms, fecal streptococci, sulphate-reducing clostridi. Total microbial counts were evaluated at 22°C and 37°C. Water temperature and residual free and totalchlorine were determined at the time of sample collection.

Briefly, one litre water samples were collected in sterile containers containing 0.01% sodium thiosulphate to neutralise any chlorine; they were kept at ambient temperature and protected from direct light. Water was processed on the day of collection and sample concentration was conducted using 0.45 μm cellulose membrane filters.

After concentration, the membranes were aseptically removed and placed into sterile screw-capped containers with 10 ml sterile distilled water. The material of the filter membrane was vortex-mixed (3 times for 30 sec).

Then, a 0.1 ml of each sample was inoculated onto two GVPC-selective agar, a medium containing cycloheximide, glycine, polymyxin B and vancomycin.

The remaining 9.9 ml was centrifuged at 3,000 R/min for 20 minutes; all the supernatant was removed but 1 ml; then, the sediment was resuspended and incubated at 50°C in a water bath for 30 min; afterwards, 0.1 ml was spread on duplicate plates of GVPC agar. These plates, laid into candle jars, were incubated at 36–37°C in a humidified atmosphere with <5% CO2.

Plates were evaluated for 10 days before reporting them negative.

Gram stain was performed in case of suspected colonies; weakly staining, gram-negative bacilli were observable. Suspected colonies with a mottled surface or an iridescent and faceted cut glass appearance, were counted from each sampling. All colonies from plates with ≤10 and 10–20 random colonies were subcultured on buffered charcoal yeast extract (BCYE) agar (with cysteine), charcoal yeast extract agar (CYE Agar Base – Oxoid), blood-agar and McConkey agar plates.

These plates were incubated at 37°C in a humidified environment for ≥2 days. Only colonies grown on BCYE were subsequently identified by two agglutination tests (i.e., *Legionella *Latex Test, Oxoid and Legionella Immune Sera "Seiken", Denka Seiken Co. LTD). The first test allows a separate identification of *L. pneumophila *serogroup 1 and serogroups 2–14 and detection of seven *Legionella *species (*Legionella *spp. group), which have been implicated in human disease: *L. longbeachae *1 and 2, *L. bozemanii *1 and 2, *L. dumoffii*, *L. gormanii*, *L. jordanis*, *L. micdadei*, *L. anisa*. The second test identifies *Legionella pneumophila *serogroup 1, 2, 3, 4, 5, 6 and *L. bozemanii*, *L. dumoffii*, *L. gormanii*, *L. micdadei*.

## Results

An elevated number of samples was collected from areas and critical sites at risk (cabin where people sleep; cabin where crew sleep; showers, washbasins, kitchen sinks), considering the modes of transmission of *Legionella *infection.

A total of 34 (37.8%) samples of 90 were collected from cabins where people sleep, 32 (35.6%) from cabins where crew sleep, 16 (17.8%) from kitchens, 6 (6.7%) from coffee bars and 2 (2.2%) from air conditioning system rooms (table 1); a total of 44 (48.8%) samples of 90 were drawn from showers, 22 (24.5%) from washbasins, 22 (24.5%) from kitchen sinks and 2 (2.2%) from evaporative condensers of air conditioning systems (table 1).

*Legionella *spp. was identified in all but one ferries (66% of the total ships), while cruiser ships were not contaminated. An average of four samples (range, 0–10) was positive per ship.

A total of 38 (42.2%) water samples of 90 were contaminated by *Legionella *spp. (table 1): 50% (17/34) collected from cabins where people sleep, 53.1% (17/32) from cabins where crew sleep, 12.5% (2/16) from kitchens, 33.3% (2/6) from coffee bars; it was not identified in water samples collected in the air conditioning system rooms.

Positive samples were drawn from showers (24/44; 54.5%), mainly those located in cabins where crew sleep, from washbasins (10/22; 45.5%), mainly those located in cabins where people sleep (60%), from kitchen sinks (4/22; 18.2%), mainly those located in coffee bars (33.3%).

**Figure 1:**
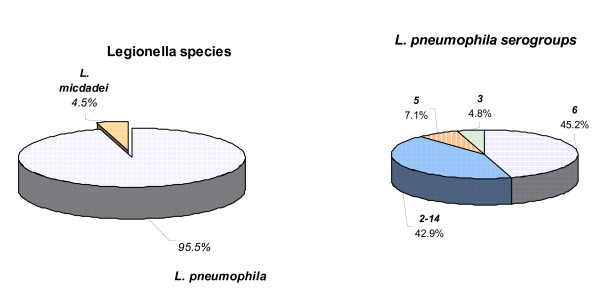
*Legionella *identification: species and serogroups.

*L. pneumophila *was isolated in 42/44 samples (95.5%), followed by *L. micdadei *(4.5%). (figure 1)

Strains were identified as *L. pneumophila *serogroup 6 (45.2%; 19 samples), 2–14 (42.9%), 5 (7.1%) and 3 (4.8%). (figure 1).

*Legionella *spp. load was high: 22.2% contained 1 × 10^3 ^– 1 × 10^4 ^Legionella per litre, while 50% 1 × 10^4 ^– 1 × 10^5^, 22.2% 1 × 10^5 ^– 1 × 10^6^, 5.6% 1 × 10^5 ^– 1 × 10^6^. Then, 27.8% contained > 100,000 Legionella per litre which is judged the critical level for environmental decontamination in absence of Legionnaires' disease, according to Italian guidelines.

Detection of microbial indicators of faecal contamination and total microbial counts at 22°C and 37°C were performed (25); bacterial load wasvariable ranging from 0 to 1,000 CFU/ml and from 0 to 784 CFU/ml at 37°C and 22°C, respectively, independently of *Legionella *spp. growth; it was demonstrated no link between microbial count and the presence of *Legionella *spp. in water samples. In only one sample microbial counts were 230 and 768 CFU/100 ml at 37°C and 22°C, respectively, in wich total and fecal coliforms were isolated (30 and 8 CFU/100 ml, respectively) but not *Legionella *spp..

**Figure 2:**
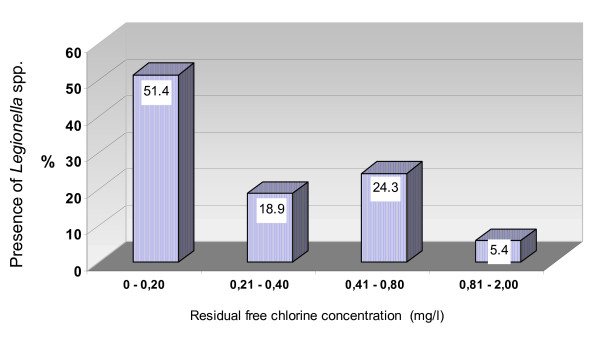
Relation between residual free chlorine concentration and presence of *Legionella *spp. in water samples.

We examined the relation between residual free chlorine and *Legionella *spp.: 0–0.2 mg/L residual free chlorine concentration was associated to a contamination of the 50% of the water samples, 0.81–2 mg/L to 5.4%; *Legionella *spp. did not significantly decrease in association to a residual free chlorine concentration of 0.21–0.80 mg/L (figure 2).

Water temperature of *Legionella *spp. positive samples ranged from 20°C to 40°C (50% ranging from 20°C to 30°C and 50% ranging from 31°C to 40°C); *Legionella *spp. was not identified in water samples whose temperature ranged from 41°C to 60°C.

Of the 90 water samples, six (6.6%), collected in a single cruise-ship, had temperature ranging from 42.4 to 56.6°C. Negative cultures of these water samples might be due to their residual free and total chlorine, ranging from 1.80 to 1.95 and from 1.90 to > 2, respectively.

## Discussion

*Legionella *is an ubiquitous bacterium that could create problems for public health; most cases of Legionnaire's disease are sporadic but nosocomial or community outbreaks can occur, such as those related to tourist accommodations [12-15].

We conducted a prevalence study in order to evaluate the presence of *Legionella *spp. in passenger vessels, i.e. ferries and cruise ships, because of the increased navigation traffic and poor national and international medical reference.

We analysed 2 cruise ships and 7 ferries; *Legionella *spp. was identified in 6/7 ferries. Microbial load was predominantly high (> 10^4 ^CFU/L or ranging from 10^3 ^to 10^4 ^CFU/L).

However, we did not identify *Legionella pneumophila *serogroup 1, which is responsible for more than 90% of clinical cases.

This finding might be related to differences in environmental prevalence among *Legionella pneumophila *serogroup 1 and non-*pneumophila *species. Several reports, evidenced that the high frequency of *Legionella pneumophila *serogroup 1 isolation from clinical samples is not strictly linked to environmental predominance but might be due to higher infectivity or more efficient intracellular growth. The low incidence of non-*pneumophila *species among clinical isolates associated to their high environmental frequency implies that these species are less virulent and pathogenic than *Legionella pneumophila *serogroup 1 [16].

It is matter of concern when passengers are subjects at risk (smokers, older people, immunocompromised individuals, etc.); *Legionella *spp., in fact, is an opportunist par excellence, that can survive in freshwater systems; high bacterial load might be an important variable related to disease's occurrence in association to long-term exposure.

Although cruise ships were *Legionella *spp.-free, ferries to Sardinia are frequently used for cruising during all seasons but summer.

Therefore, it is important to analyse the risk of acquiring infection with regard to water supplies and hosts in order to provide preventive interventions.

According to recent Italian guidelines targeted to administrators of hotels and thermal baths, contamination level is high in 77.8% of our samples, requiring disinfecting measures and later evaluation, regardless of disease's occurrence; the remaining samples (22.2%) have to be surveyed, requiring decontamination measures if one or more cases of legionellosis are identified [10].

Prophylactic measures, as stated by EWGLI, imply that all water services should be routinely checked for temperature, water demand and inspected for cleanliness and use [8].

Principal items include the reduction of the possibility of using contaminated water by disinfecting (chemical disinfection), filtering and suitably storing the water; keeping cold water cold and hot water hot; avoiding the dead ends in pipes; properly cleaning and disinfecting spas; periodically cleaning or replacing the devices that might promote dissemination of Legionella.

## Conclusion

The absence of a correct prevention of Legionella infections onboard ship might cause a lot of medical – sanitary, legal and economic problems (deaths, negative impact on tourism, possible removal of the ship from the service and suspension of activity of its crew for many days, etc.) [8,13,17].

Furthermore, environmental decontamination is useful as punctual action but does not lead to a definitive solution to the problem.

Therefore, it is important to identify a person responsible for supervision and health safety in order to prevent or to control the risk from exposure, according to EWGLI and Italian guidelines; the person has to conduct an assessment of the sites at risk in the water systems present in the premises and to implement precautions for controlling any identified risk from *Legionella *bacteria. He has to be competent to assess the risks of exposure to Legionella bacteria in the water systems present in the premises and the control measures (e.g. a microbiologist, environmental health officer or water engineer with is specific expertise).

This aspect might be fundamental in Sardinia, an island devoted to tourism, where prevention of infectious disease acquisition from environmental sources and healthcare have to be associated to elevated level of quality of dwelling places.

Shipping companies have a keen interest in effects of our epidemiological study, showing willingness to proceed with investigation targeted to *Legionella *bacterium and to verify the efficacy of decontamination performed after the survey.

## Abbreviations

CFU: colony forming unit

WHO: World Health Organization

EWGLI: European Working Group for *Legionella *Infections

## Competing interests

The author(s) declares that he has no competing interests.

## Authors' contributions

All of the authors participated in planning and design of the study, and all read and approved the manuscript. MD and MGD participated to the waters analysis. AA, AP, GS, MDM, BMA and EM conceived the study, participated in its design and wrote the manuscript.

## note

Table 1 – Distribution of *Legionella *spp. in ferries and cruise ships.

## Pre-publication history

The pre-publication history for this paper can be accessed here:
